# Single-step Precision Genome Editing in Yeast Using CRISPR-Cas9

**DOI:** 10.21769/BioProtoc.2765

**Published:** 2018-03-20

**Authors:** Azat Akhmetov, Jon M Laurent, Jimmy Gollihar, Elizabeth C Gardner, Riddhiman K Garge, Andrew D Ellington, Aashiq H Kachroo, Edward M Marcotte

**Affiliations:** 1 Center for Systems and Synthetic Biology, Institute for Cellular and Molecular Biology, University of Texas at Austin, Austin, TX, USA; 2 Institute for Systems Genetics, Department of Biochemistry and Molecular Pharmacology, New York University Langone Health, New York, NY, USA; 3 The Department of Biology, Centre for Applied Synthetic Biology, Concordia University, Montreal, QC, Canada; 4 Department of Molecular Biosciences, University of Texas at Austin, Austin, TX, USA

**Keywords:** CRISPR, Homologous recombination, Humanization, Ortholog complementation, Genome editing, Yeast engineering

## Abstract

Genome modification in budding yeast has been extremely successful largely due to its highly efficient homology-directed DNA repair machinery. Several methods for modifying the yeast genome have previously been described, many of them involving at least two-steps: insertion of a selectable marker and substitution of that marker for the intended modification. Here, we describe a CRISPR-Cas9 mediated genome editing protocol for modifying any yeast gene of interest (either essential or nonessential) in a single-step transformation without any selectable marker. In this system, the Cas9 nuclease creates a double-stranded break at the locus of choice, which is typically lethal in yeast cells regardless of the essentiality of the targeted locus due to inefficient non-homologous end-joining repair. This lethality results in efficient repair via homologous recombination using a repair template derived from PCR. In cases involving essential genes, the necessity of editing the genomic lesion with a functional allele serves as an additional layer of selection. As a motivating example, we describe the use of this strategy in the replacement of HEM2, an essential yeast gene, with its corresponding human ortholog ALAD.

## Background

*Saccharomyces cerevisiae* (Baker’s yeast) has a long history as a genetically tractable organism, and there are an array of methodologies to manipulate the yeast genome. However, until recently it has been necessary to apply selection to isolate clones possessing the desired genetic alteration (Kearse *et al*., 2012; DiCarlo *et al*., 2013; Lee *et al*., 2015; Kachroo *et al*., 2017). In cases where arbitrary, scar-less editing of the genome is desired, the solution is typically a two-step process: First a selectable cassette (containing the URA3 marker, for example), flanked by homology arms targeting the region of interest, and sometimes containing nuclease targeting sites (*i.e*., I-SceI sites) to aid in the removal of the cassette at the later stage, is knocked in via homologous recombination (HR). The small subpopulation of successful integrants is isolated by selecting for the cassette. Second, the marker is eliminated through highly efficient sequence specific methods such as site-specific recombination or endonuclease cleavage (I-SceI) to generate the desired form of the edited genomic locus. Two steps are necessary because no method was available which is both scar-less and efficient enough such that no selection is required.

The development of CRISPR/Cas9 technology in yeast has eliminated the need for this two-step process. Cas9 efficiently creates double-stranded breaks (DSBs) in yeast DNA at virtually any arbitrary locus–provided a PAM sequence is proximal to the desired cut site. When an appropriate repair template is provided, these DSBs are repaired through the endogenous HR system of yeast. Cas9 directed to the desired genomic locus via the guide RNA sequence creates double-stranded break (DSB) in the genome. The CRISPR target site is retained in cells which fail to repair the target site as expected, which allows Cas9 to repeatedly cleave the same region until HR-mediated editing takes place. Rarely, non-homologous end-joining (NHEJ) can generate mutations which block Cas9 cleavage despite failing to incorporate the expected genomic alterations. More commonly, cells simply succumb to the stress of repeated Cas9-induced genomic cleavages. In an appropriately conducted experiment, the majority of the surviving population tends to be cells which have lost their CRISPR target site by incorporating the desired genomic alteration via HR. Cas9 thus acts as a counter-selection acting directly on genomic sequence, rather than its phenotypic manifestations.

Here, we use an approach developed by Dueber and colleagues (Lee *et al*., 2015) to rapidly generate single, self-contained plasmids that express both the Cas9 nuclease and guide RNA required for targeting a desired locus. These plasmids, when co-transformed with an appropriate repair template provided as a linear PCR product, allow efficient, precise, single-step replacement of any arbitrary yeast gene with an introduced sequence of interest. Only selection for the Cas9 and gRNA-expressing plasmid is required, which tends to select for correct genomic modification by proxy due to efficiency of targeting and repair. This strategy was used extensively in our ortholog complementation research (Kachroo *et al*., 2017) to rapidly humanize, bacterialize and plantize many essential yeast genes. A CRISPR based approach is uniquely suited to this case, because it strongly encourages HR with functional alleles. False positives, arising from CRISPR sites being mutated by NHEJ without incorporation of a new allele, are minimal because they are often not viable. Additionally, disruption of the target gene’s function is brief, eliminating the need for constructing and maintaining a complementing plasmid to sustain yeast through an otherwise lengthy engineering process. Further, given that CRISPR selects against sequence regardless of function, it is still possible and practical to alter non-essential genes (or even non-genic regions) with this technique; indeed, we have reported successful humanization of the nonessential yeast gene HEM14 with this method (Kachroo *et al*., 2017) and we have used this system to incorporate site-directed changes in proteins with high efficiency.

## Materials and Reagents

1. Pipette tips (Mettler Toledo, catalog numbers: 17005872, 17005874, 17007089)
2. 96-well plate (VWR, catalog number: 82006-636)
3. 0.2 μm filter (Fisher Scientific, catalog number: 09-719C)
4. Petri plates (VWR, catalog number: 25384-342)
5. Yeast (BY4741)
6. MoClo Yeast Toolkit (YTK, Addgene kit, Addgene, catalog number: 1000000061). Toolkit includes plasmids pYTK050, pYTK003, pYTK072, pYTK083, pYTK036, pYTK008, pYTK047, pYTK073, pYTK074, pYTK081 and pYTK084
7. PCR template for the sequence which will replace the target gene (*e.g*., cDNA, plasmid-based clone, *etc*.)
  Note: For demonstration purposes, this protocol will assume replacement of S. cerevisiae HEM2 with its human ortholog ALAD.
8. NEB 5-alpha Competent *E. coli* (New England Biolabs, catalog number: C2987)
9. DNA stain (Thermo Fisher Scientific, Invitrogen™, catalog number: S33102)
10. T7 ligase (New England Biolabs, catalog number: M0318S)
11. T4 ligase buffer (New England Biolabs, catalog number: B0202S)
12. Restriction enzymes BsaI (New England Biolabs, catalog number: R0535S) and BsmBI (New England Biolabs, catalog number: R0580S)
13. LB plates with antibiotic selection
  - Ampicillin (Sigma-Aldrich, Roche Diagnostics, catalog number: 10835242001)
  - Spectinomycin (Sigma-Aldrich, catalog number: PHR1426)
14. Chloramphenicol (Sigma-Aldrich, catalog number: C0378)
15. High-fidelity DNA polymerase for repair template PCR, such as KAPA HiFi (Kapa Biosystems, catalog number: KK2601)
16. Zymo DNA Clean&Concentrator-25 kit (Zymo Research, catalog number: D4005)
17. Zymo EZ yeast transformation II kit (Zymo Research, catalog number: T2001)
18. Optional: 100 mM lithium acetate can be used in place of EZ 1 solution from the EZ competent yeast cell kit. (Lithium acetate can be obtained from Sigma-Aldrich, catalog number: L6883)
19. Accuprime Pfx (Thermo Fisher Scientific, Invitrogen™, catalog number: 12344024)
20. Optional: 5-fluoroorotic acid (Sigma-Aldrich, catalog number: F5013), if counter-selection will be used (see Procedure E)
21. D-Sorbitol (Sigma-Aldrich, catalog number: S3889)
22. Zymolyase (MP Biomedicals, catalog number: 320921)
23. LB Broth, Lennox (BD, catalog number: 240210)
24. YPD powder (BD, catalog number: 242820)
25. Agarose (Thermo Fisher Scientific, Invitrogen™, catalog number: 16500500)
26. Agar (SERVA Electrophoresis, catalog number: 11396)
27. Yeast nitrogen base without amino acids (BD, catalog number: 291940)
28. Ammonium sulfate (Sigma-Aldrich, catalog number: A4418)
29. Dextrose (Avantor Performance Materials, catalog number: 1919)
30. SC-Ura dropout powder (Sigma-Aldrich, catalog number: Y1501)
31. Zymolyase solution (see Recipes)
32. Lithium acetate (see Recipes)
33. LB medium (see Recipes)
34. YPD agar plates (see Recipes)
35. SD-Ura agar plates (see Recipes)

## Equipment

1. Thermocycler (Bio-Rad Laboratories, catalog number: 1861096)
2. Light source for visualization of DNA stain (Thermo Fisher Scientific, Invitrogen™, catalog number: G6600)
3. 12-channel pipette (Mettler Toledo, catalog number: 17013810)
4. Standard gel electrophoresis tank and accessories (Bio-Rad Laboratories, catalog number: 1640302)
5. Autoclave

## Software

1. Geneious v8.0 (Kearse *et al.*, 2012) or higher, to design gRNA and repair template (replacement gene). Other gRNA design software can be used as well, such as E-CRISP (Heigwer *et al.*, 2014)
2. BLAT (Kent, 2002)

## Procedure

### A. Preparation of CRISPR plasmid (for a diagrammatic overview of the cloning process, see Figure 1)

**Figure 2:**
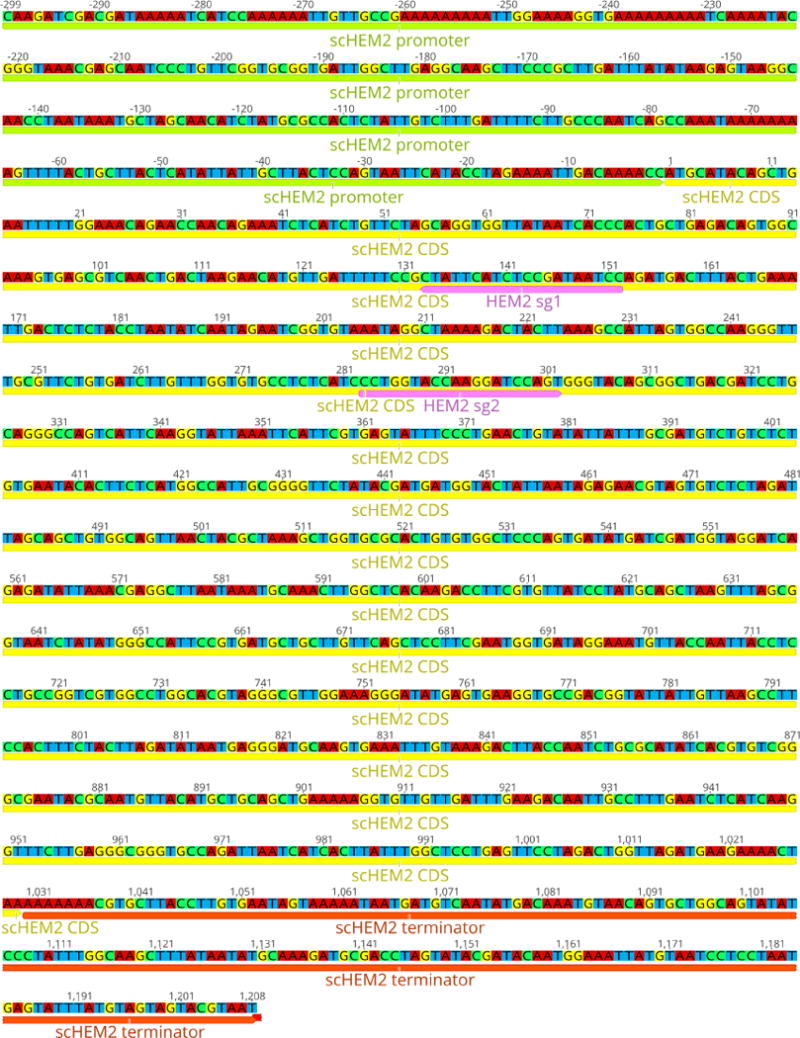
Diagram of the native yeast HEM2 locus, showing positions of the example guide RNAs sg1 and sg2

1. Design two guide RNA (gRNA) sequences targeting the open reading frame (ORF) for the yeast gene to be replaced using Geneious, or a similar tool such as E-CRISP (Heigwer *et al.*, 2014).
  - gRNA sequences can often have low activity in practice, despite being predicted to be highly efficient by software tools. In order to minimize setbacks due to a gRNA which turns out to function poorly, we advise designing multiple gRNAs from the outset, and taking them through the cloning steps in parallel, up to and including the construction of the CRISPR plasmids. Both plasmids should then be tested for their ability to target the yeast genome and kill cells (described in later steps) to empirically determine and confirm their activity.
  - We have not noticed a strong effect of the location of the gRNA within the ORF. During homologous repair, DNA can be resected up to several kilobases from the break site (Mimitou and Symington, 2009; Chen *et al*., 2011), so the gRNA need not be very close to either terminus of the ORF. It is however important to select a gRNA such that the target site is not present after replacement (*i.e*., the gRNA should target the yeast ORF, but not the replacement gene).
  - Example: For targeting HEM2, the sequences GGATTATCGGAGATGAATAG (‘sg1’, on the non-coding strand) and CCTGGTACCAAGGATCCAGT (‘sg2’, on the coding strand) were predicted to have high activity (see Figure 2).
2. Order forward and reverse oligonucleotides with the gRNA sequence and Golden Gate compatible overlaps:
  - Forward oligo consists of the 5′ insert GACTTT followed by the 20 bp guide sequence specific to the target gene. Example forward oligo for HEM2 sg1 (underline indicates 5′ Golden Gate overhang): GACTTTGGATTATCGGAGATGAATAG.
  - Reverse oligo consists of the 3′ insert AAAC, followed by the reverse complement of the 20 bp guide sequence, followed by AA, which complements part of the GACTTT insert on the forward oligo. Example reverse oligo for HEM2 sg1 (underline indicates 3′ Golden Gate overhang): AAACCTATTCATCTCCGATAATCCAA.
3. Mix forward and reverse oligos (50 μM each) for each gRNA in a total volume of 20 μl and anneal with each other using a thermocycler with the program below. It is unnecessary to phosphorylate the insert.
  95 °C for 5 min
  55 °C for 15 min
  25 °C for 15 min
4. First Golden Gate cloning reaction to transfer into shuttle vector: Set up cloning reaction with annealed oligos and pYTK050 (Table 1).
  A 2:1 molar ratio of insert:plasmid is recommended for optimal Golden Gate cloning of linear DNA.
5. Transform the reaction into competent bacteria and plate with chloramphenicol selection (170 μg/ml). View colonies under UV light and pick the white colonies (those not showing GFP fluorescence), then grow in liquid culture and purify plasmid. The vectors used in Golden Gate reactions described in this protocol are all GFP-dropout vectors: They contain a GFP gene which will be silenced upon successful cloning. Therefore, GFP fluorescence indicates an invalid construct, while successful constructs will lose the GFP gene and the resulting colonies will be white.
  Optionally, the plasmid can be sequenced to check for errors or mutations in the gRNA sequence, such as may occur during synthesis.
6. Second Golden Gate cloning reaction to create gRNA cassette plasmid: Set up cloning reaction which includes connector plasmids ConL1 and ConRE (Table 2).
  For best efficiency, all plasmids should be present at the same molarity in plasmid-based Golden Gate assemblies.
7. Transform the reaction into competent bacteria and plate with ampicillin selection (60 μg/ml). View colonies under UV light and pick the white colonies (those not showing GFP fluorescence), then grow in liquid culture and purify plasmid.
8. Third and final Golden Gate cloning reaction to construct the yeast-compatible, complete CRISPR plasmid: Set up Golden Gate cloning reaction with connector plasmid from the previous step, and yeast –Ura backbone plasmid, and Cas9 plasmid (Table 3).
9. Transform the reaction into competent bacteria and plate with kanamycin selection (50 μg/ml). View colonies under UV light and pick the white colonies (those not showing GFP fluorescence), then grow in liquid culture and purify plasmid.

The resulting construct is a self-contained CRISPR plasmid, which when transformed into yeast will cause double-stranded breaks (DSBs) at the locus determined by the gRNA sequence cloned into it. 500 ng of this will be used for each yeast transformation, so if multiple replacements are planned, it is helpful to dilute the CRISPR plasmid to a standardized concentration for easier transformation set up later on.

### B. Preparation of repair template DNA

**Figure 3:**
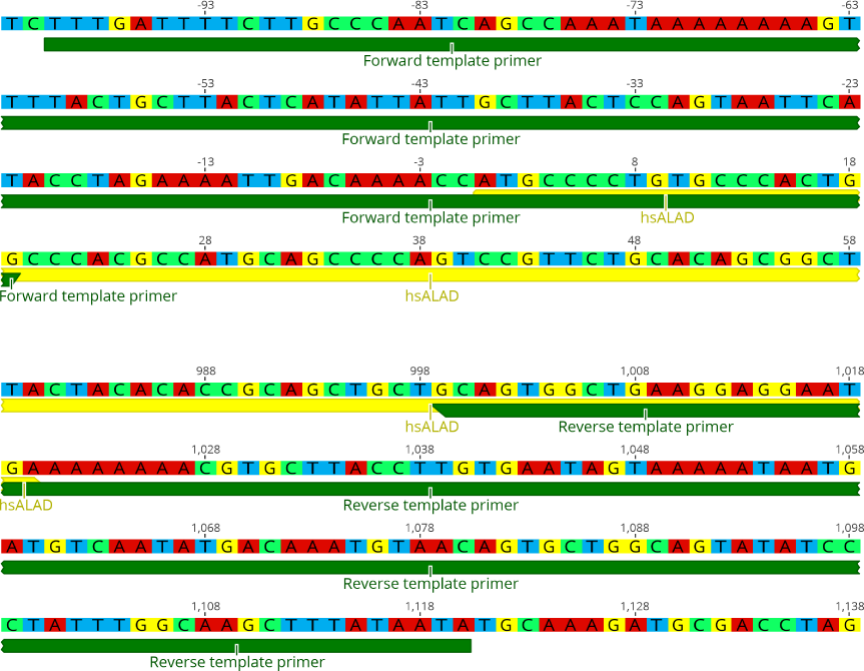
Diagrams of example template primer designs for the replacement of HEM2 with hsALAD

1. Design the template DNA using Geneious or any other cloning software. Obtain the genomic sequence of the target yeast gene (‘old gene’), and the coding sequence (CDS) of the replacing gene (‘new gene’). The CDS should not contain introns. Create a gene model for the replaced locus by editing the sequence of the old gene so that it contains the new gene in the correct position (*i.e*., the desired outcome of replacement).
  We find that replacement works best if the original yeast stop codon is left intact. Otherwise, modifying the new gene, for instance to codon optimize for yeast, has proven unnecessary.
2. Design template PCR primers which anneal to about 25 bp of the 5′ and 3′ ends of the new gene’s CDS, and also the 5′ and 3′ UTR immediately adjacent to the ORF (the homology arms). Figure 3 shows an example of primer design for replacing the yeast HEM2 gene with its human ortholog ALAD. This process is much easier using the gene model constructed in the previous step: The sequence covering the junction points between yeast genome and the new gene CDS can be used directly as primer sequence.
  - The length of the region complementary to the new gene CDS is determined only by standard PCR efficiency concerns, such as melting temperature. This area will serve as a toehold for the first few cycles of the PCR.
  - The length of the homology arms is critical for efficient replacement. We find that homologies of at least 70 bp are necessary (in which case the entire primer oligo will be about 90 bp long), and for some genes, 170 bp homologies may be necessary. For even more difficult replacements, longer homology arms can be cloned separately, but we have found that homologies longer than 500 bp are unlikely to increase efficiency further.
3. Use template PCR primers to amplify a large amount of repair template DNA using a high-fidelity polymerase.
  - We find that it is helpful to first conduct several test PCRs with different polymerases. Due to the particular design of the template primers, this PCR can sometimes run inefficiently or generate unwanted non-specific products. Different polymerases have different characteristics, and often a reaction which fails with one polymerase will run efficiently with another, rendering laborious PCR optimization unnecessary.
  - At least 5 μg of template DNA is needed per yeast transformation, which can usually be obtained from a single 50 μl PCR. Difficult replacements can often be facilitated by using more (10 μg) template DNA, and if multiple transformations are to be performed the amount will also need to be scaled up accordingly. Often several PCRs are necessary to produce enough DNA.
  - If very large amounts of template DNA are needed, or an efficient PCR is difficult to set up, an alternative method is to clone the template sequence onto a plasmid, which can be amplified in bacteria with the template DNA excised using restriction enzymes.
4. Check the template PCR with agarose gel electrophoresis.
  As long as a sufficient amount of the correct template is produced, non-specific products do not necessarily constitute a problem for the replacement. Because the non-specific products usually lack appropriate homologies, they will not be efficiently integrated into the yeast genome. However, if significant amounts of them are present, they will cause over-estimation of template DNA during spectrophotometry-based quantification; thus the amount of template DNA used in the transformation would need to be adjusted accordingly. Alternatively, the PCR can be optimized to reduce non-specific products, or only the correct product can be quantified from the gel using a DNA ladder calibrated for quantity estimation.
5. Purify template PCR using the Zymo DNA Clean&Concentrator-25 kit. Elute in double distilled water.

Ideally, the volume of DNA included in yeast transformation should be small, so as to not interfere with the transformation reagents. The elution volume should be adjusted accordingly so that the resulting concentration of DNA is not too low. In our experiments, we have found that eluting with 25 μl double distilled water will usually yield 400-800 ng/μl DNA, which is suitable for transformations.

### C. Yeast transformation

**Figure 4:**
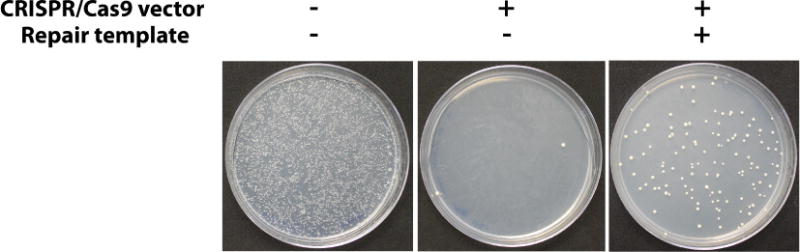
Representative assay results Yeast cells are rescued from DSB lethality (center plate) when an appropriate repair template is provided (right plate). The left plate is a negative control of cells carrying a control plasmid with the same selectable marker (URA3) done to estimate the transformation efficiency of the yeast strains being used.

**Figure 5:**
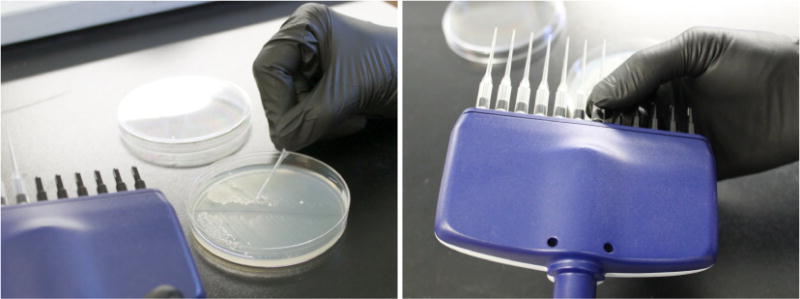
Demonstration of colony picking technique with 12-channel pipette

1. Prepare competent yeast cells using the Zymo EZ competent yeast kit according to the kit instructions.
  The EZ 1 solution in this kit can be substituted with 100 mM lithium acetate without significant change in transformation efficiency.
  The amounts given in the kit manual can be slightly modified: 2 ml yeast culture can be used to produce 100 μl of competent yeast, which is sufficient for two transformations, 50 μl each.
2. Set up a transformation reaction: Mix 50 μl competent yeast, 500 μl EZ 3 solution, 500 ng of CRISPR plasmid and 5 μg repair template DNA (up to 50 μl total volume). Incubate at 30 °C as directed by kit manual and plate on –Ura medium.
  When using a new gRNA for the first time, gRNA efficiency can be estimated with a control transformation, which is performed as stated but without repair DNA. When the CRISPR plasmid is introduced without a repair template, it will repeatedly cleave the target locus, causing toxicity. Very few or no colonies are the ideal outcome, since this indicates highly efficient CRISPR cleavage and low background rate. Cells can survive the CRISPR plasmid uptake without repair DNA if the CRISPR activity is stochastically low (such as due to poor gRNA efficiency) or mutations at the CRISPR target locus can be tolerated (which produces false transformants even in presence of the repair template).
3. When colonies appear on the –Ura plates, collect up to 12 of them with a pipette tip and suspend in 50 μl water. These suspensions will be screened for confirmed replacements. Yeast suspensions can be stored at 4 °C and used to start new cultures for up to 2 weeks.
  - Typically, colonies will appear on –Ura plates (Figure 4) after 1-3 days. In some cases, the replacement will impose a significant fitness defect such that up to 6 days may be required for colonies to appear, but we have not encountered cases where colonies from a successful transformation take longer than 6 days to grow.
  - The uracil dropout medium will select against cells which failed to take up the CRISPR plasmid (which confers uracil prototrophy), but because the CRISPR plasmid is toxic to cells unless a successful replacement occurs (eliminating the CRISPR target locus) only cells which have a replaced locus are expected to survive. However, due to spontaneous hypoactivity of the CRISPR system, mutations in the CRISPR target locus (DiCarlo *et al*., 2013), and cells which manage to survive CRISPR-associated DSBs, there will be a background rate in the form of false transformant colonies which do not carry the correct genomic replacements. To save time, we recommend collecting several transformant colonies and screening them in parallel.
  - To streamline this process (especially when several replacements are performed in parallel), pick colonies with pipette tips and manually attach them to a multichannel pipette (Figure 5). The multichannel pipette can then be used to suspend all 12 samples in one row of small PCR tubes or a 96-well plate.

### D. Colony screening via PCR

1. Design confirmation PCR primers: Primer pairs should be selected such that the forward primer anneals to the yeast UTR while the reverse primer anneals only to the new gene CDS but not the old gene’s ORF. Thus, the product should span the junction point between foreign sequence and native yeast genome. The yeast UTR primer should preferably not overlap the homology region.
  - Ideally, the product size should be small, about 300 bp, for a faster and more robust PCR.
  - It is sufficient to check only the 5′ junction point, since it is rare for integration to proceed as expected at one end of the gene but introduce artifacts at the other.
  - If desired, the absence of the yeast ORF can also be tested by using a reverse primer which anneals to yeast ORF only. However, lack of product from such a primer pair is not sufficient to confirm a clone, since the reaction is liable to fail for unrelated reasons (such as poor lysis of cells).
2. Prepare lysates of harvested transformants: Mix 5 μl of each yeast suspension with 15 μl zymolyase solution.
3. Incubate lysates for 30 min at room temperature, then 15 min at 37 °C and 5 min at 95 °C.
4. Set up 20 μl colony PCRs with confirmation primers and using Accuprime Pfx as the polymerase. Use 1 μl of the lysate as template DNA.
  - We find that other polymerases do not perform well due to impurities from the yeast lysates.
  - Due to the impurities introduced by the lysate, the colony PCR may spontaneously fail, leading to false negatives. To ameliorate this problem, a positive control PCR can be performed for each lysate, which is identical to the confirmation PCR but uses primers complementary to an unrelated, unmodified locus in the genome. We use two primers targeting a 500 bp segment of the yeast ERG13 promoter for this purpose (forward CGAACTGGATGAGATGGCCG and reverse CATGCTGCACCTTTTATAGTAATTTGGC).
5. Check the colony PCRs for product by agarose electrophoresis. Lysates from clones with the correct modifications should generate a product with the confirmation primers. Background false transformants (*e.g*., mutants) will not produce a band.
  - A PCR product from the confirmation primers is sufficient evidence of successful integration of the repair template. For further verification, the locus can be sequenced, but we have found that dramatic sequence artifacts rarely occur in clones confirmed by PCR, the most common mutations are single-basepair substitutions or indels, which typically constitute a minority of confirmed clones.
  - Lack of product from the confirmation primers is inconclusive per se. In such cases, it is worthwhile to consider additional evidence, such as whether the positive control PCR worked (if not, the lysis may have failed).
6. Confirmed clones can be propagated by starting a new culture from the original suspensions of yeast in water.

### E. Curing of the CRISPR plasmid

1. Streak original water suspensions of confirmed clones on YPD. The CRISPR plasmid is low copy and can be spontaneously lost in absence of selection.
2. Pick 10 colonies from the YPD plate and patch each one on YPD and SD-Ura plates.
3. Incubate both plates, and collect cells from patches which grew only on YPD but not on SD-Ura. Isolates which still carry the CRISPR plasmid will grow on uracil dropout medium, but those which have lost the plasmid will not. Typically, 3 days is sufficient to confirm lack of Ura prototrophy, but if slow growth on uracil dropout is suspected, incubation can be extended to up to 6 days to definitively confirm no growth on uracil dropout.
  The plasmid can also be cured by counterselecting on 5-fluoroorotic acid (FOA) plates (Boeke *et al.*, 1987). However, there is a possibility that this FOA method will generate some colonies that are not cured of the plasmid but rather have acquired a mutation in the Ura marker (thus continuing to express the gRNA). Thus, FOA counterselection should not be used (as opposed to replicate patches on YPD and –Ura) if it is important to ensure curing of the plasmid, rather than simply abrogating Ura prototrophy. On the other hand, the FOA method can save time if only loss of –Ura heterotrophy is desired, for instance to enable a subsequent transformation with a different Ura-selectable plasmid.

## Data analysis

The data analysis needs for this procedure are minimal. Most importantly, when using Geneious to design gRNA sequences, it is desirable to select gRNA sequences that have high predicted on-target activity (automatically calculated by Geneious). gRNA sequences with high predicted activity may have low actual activity, but they will be less likely to exhibit low activity than sequences with low predicted activity. The distance of the gRNA target site can be up to 1 kb away from either homology region without perceptible negative consequence, thus gRNAs should be selected primarily based on high activity rather than location (provided that they lie between the two homology arms).

## Notes

1. We have found that even among gRNAs with high predicted activity, some will fail to induce double-strand breaks with sufficient efficiency for editing. It is highly recommended that for each target locus, several gRNA are designed and tested in parallel, to ensure that at least one will be a sufficiently good DSB inducer for purposes of genome editing.
2. If a given gRNA exhibits significant off-target activity, the likely outcome is that off-target cleavage will kill most of the transformed yeast cells. Successful, efficient genome editing in yeast relies on lethality associated with DSBs at the target locus being rescued by HR (allowing efficient repair of the DSB) and abrogation of the gRNA target site (preventing further cleavage). In the event off-target activity, HR may likely not take place because no repair template with homology to the off-target site has been supplied, moreover the gRNA site will not be eliminated for the same reason. Further, the confirmation strategy we suggest is such that only repair at the correct locus will produce a positive result. However, it is nevertheless worthwhile to ensure that selected gRNA target sites do not occur at other locations in the genome, where cleavage is not intended. Although it is very unlikely for the combined 23 bp target sequence to appear multiple times in the yeast genome, we recommend confirming that candidate gRNA sites appear only in the target locus using a tool such as BLAT.
3. gRNA targets consist of a 20 bp sequence (which will also be included in sgRNA sequence and become part of the Cas9 complex) followed by a 3 bp PAM sequence (which takes the form of NGG for Cas9 described in this protocol). The PAM sequence does not become part of the gRNA, but it must be present in the target genome for Cas9 cleavage to occur. This can be verified by attempting to align the gRNA sequence to the sequence of the repair template– typically, CRISPR activity will be very low with more than 5 mismatching basepairs, although mismatches in the PAM and proximal to the PAM appear to have more significance (Kuscu *et al.*, 2014). When replacing with very similar sequences, such that it is difficult to find good gRNA sites unique to the target locus, one strategy that can be adopted is to introduce synonymous mutations in the repair template sequence which alter the PAM site or PAM-proximal nucleotides. Alternatively, recent research suggests that using shorter gRNA may increase specificity, since the 8-17 PAM-proximal nucleotides contribute disproportionately to CRISPR target recognition (Xu *et al.*, 2017).
4. There is some variability in the yeast transformation step, and depending on how the competent cells were prepared, and how the transformation was performed. Most commonly, the number of resulting colonies will vary somewhat between transformations of identical strains with identical reagents, but usually this variation will be less than tenfold. When a transformation produces a fair number of colonies (at least 10) yet none of them are found to be correct clones upon screening, simply repeating the transformation is unlikely to improve results. The most straightforward avenues of increasing the number of correct clones are to increase the amount of repair template DNA, and to produce repair template DNA with longer homologies.
5. If no colonies appear after transformation, the reason may be low transformation efficiency. In this case, several troubleshooting steps can be taken (described in detail in the documentation of the Zymo EZ competent yeast kit). We have found the following to be effective:
  - Thoroughly vortexing the mixture of competent cells and DNA.
  - Longer incubation time for the transformation (1.5 h instead of the 45 min).
  - Including more cells in the transformation.
  - Competent cells seem to perform slightly better when frozen once (slowly in -80 °C) than freshly prepared cells.
6. When the CRISPR reagents and repair template are transformed into yeast cells, the resulting transforming colonies will be of three kinds with respect to the targeted locus:
  - Correct transformants which bear the sequence of the repair template.
  - False transformants which bear the original, unedited sequence.
  - Mutants.
7. In our experiments, we have found that the first two classes predominate unless mutants are specifically selected for. Even in the absence of a repair template, the majority of false transformants will not be mutants. Due to the efficient HR system of *S. cerevisiae*, if the conditions of the experiment are adequate then editing will take place at a very high rate. Thus, typically, the proportion between the first two of the three classes listed above will be such that the transformants are either mostly correct or all false. The third class, or mutants, we have found to be very rare in either case unless specifically selected for. As a consequence, it is rarely necessary to screen a very large number of colonies to determine whether an editing experiment has succeeded. However, it is desirable to collect several confirmed clones to minimize issues caused by artifacts, such as mutant edited sequence caused by errors during PCR (with the reagents and protocols described in this text, we have found clones with mutant edited sequence also be very rare).
8. Selecting yeast transformants with a single amino-acid dropout medium is normally a straightforward process, and colonies can be seen within 1-2 days of plating. However, occasionally the genome editing process itself, or the resulting edited sequence, can result in a growth defect in the resulting cells. Thus, if no colonies appear, incubating the plate for a longer period can produce colonies. In the most extreme case we observed, it took 6 days for colonies to appear on a uracil dropout medium, but several clones were later confirmed by PCR and sequencing; these clones consistently exhibited slow growth in subsequent culture on rich medium (YPD) as well.
9. Some combinations of target locus and repair template may lead to a mixture of large and small yeast colonies after transformation. If this occurs, generally it is best to screen an adequate number of colonies for each size class. It may be that the correct edits create much slower growing strains, thus the large colonies are false while the small ones have the desired edit. Conversely, if the desired sequence does not interfere with normal growth, but mutations arising from NHEJ do, then larger colonies will tend to be the correct clones. We have observed examples of either case when humanizing and bacterializing various loci. It is difficult to predict a priori which case will be evident for a given transformation, therefore it is often more practical to screen colonies and recording their size, and also ensuring that each size is adequately represented in the screen.
10. When picking colonies for the colony PCR screen, only a small quantity of cells is needed. Most likely as little as 1,000 cells will be sufficient to obtain a PCR product. We have often chosen to collect slightly larger numbers of cells to visually confirm their suspension in water by turbidity. However, too many cells lead to incomplete lysis and inhibition of the colony PCR. With cell clumps larger than 1-2 mm the colony PCR will often fail. So ideally, the cells collected from the colony should form only a tiny speck, 0.5 mm or smaller in diameter. It is helpful to include the positive control PCR when screening, to identify samples which failed to produce a PCR product due to poor lysis. Lysis and PCR can be repeated for these samples if needed.
11. It is possible to adapt the protocol described here for the simultaneous replacement of multiple genes. The Mo Clo toolkit allows for cloning up to 4 different gRNA cassettes on the same CRISPR plasmid; for this, the gRNAs would be captured on pYTK050 as described here, but in the second Golden Gate reaction, instead of the ConL1 and ConRE plasmids, the first gRNA would be cloned with ConL1 and ConR2, the second with ConL2 and ConR3, the third with ConL3 and ConR4 and the fourth with ConL4 and ConRE (this process is explained in detail in Lee *et al.*, 2015). All of these cassette plasmids would then be included in the final Golden Gate reaction to assemble the CRISPR plasmid. Then, during transformation of yeast, templates for each of the included gRNAs will need to be co-transformed. However, multiple replacements are even more dependent on efficient transformation, cleavage and repair than single replacements, and some additional work may be necessary to optimize these parameters in practice.

## Recipes

1. Zymolyase solution (50 ml)
  - Weigh 9.11 g D-sorbitol
  - Dissolve in 50 ml distilled, deionized water to make 1 M sorbitol and autoclave
  - Weigh 0.25 g zymolyase and dissolve in sorbitol solution
  - Aliquot and store at -20 °C
2. Lithium acetate, 100 mM (40 ml)
  - Weigh 0.408 g lithium acetate dehydrate
  - Dissolve in 40 ml distilled, deionized water
  - Filter sterilize (0.2 μm filter) and store at room temperature
3. LB medium (1 L)
  - Weigh 25 g LB powder
  - For solid medium, add 15 g agar
  - Dissolve in distilled, deionized water for 1 L total volume
  - Autoclave and let it cool to 60-70 °C
  - Pour in Petri plates so that the medium covers the visible area of the plate
  - Let plates cool and solidify at room temperature, store at 4 °C
4. YPD (1 L)
  - Weigh 50 g YPD powder
  - For solid medium, add 20 g agar
  - Dissolve in distilled, deionized water for 1 L total volume
  - Autoclave and let it cool to 60-70 °C
  - Pour in Petri plates so that the medium covers the visible area of the plate
  - Let plates cool and solidify at room temperature, store at 4 °C
5. SD-Ura (1 L)
  - Weigh 1.5 g yeast nitrogen base w/o amino acids, 5 g ammonium sulfate, 20 g dextrose, 2 g SC-Ura dropout powder
  - For solid medium, add 20 g agar
  - Dissolve in distilled, deionized water for 1 L total volume
  - Autoclave and let it cool to 60-70 °C
  - Pour in Petri plates so that the medium covers the visible area of the plate
  - Let plates cool and solidify at room temperature, store at 4 °C

## Figures and Tables

**Figure 1:**
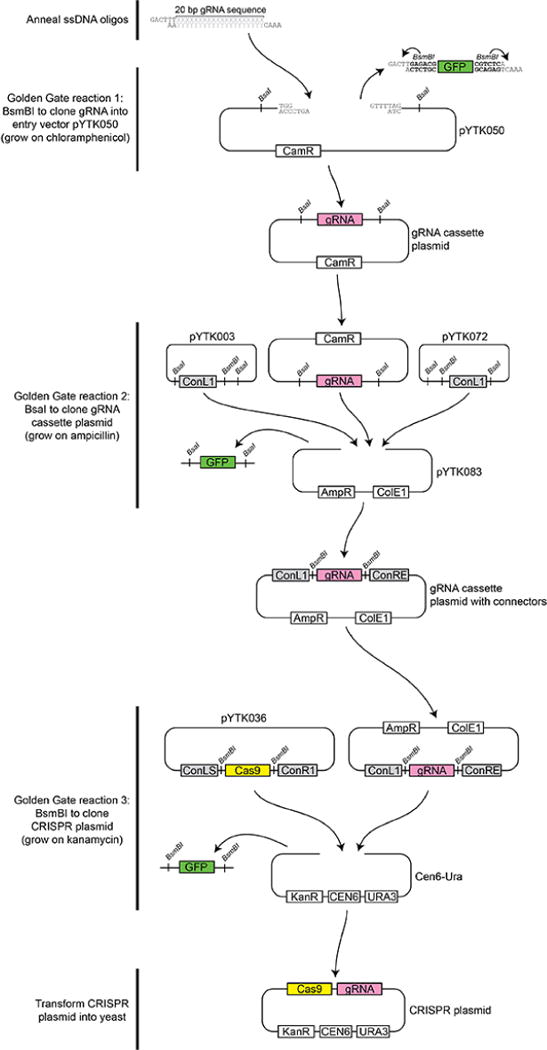
Overview of the CRISPR plasmid construction process In the first step Xs and Ys represent the gRNA sequence selected, and BsmBI recognition site is indicated in bold.

**Table 1:** Golden Gate reaction for cloning into shuttle vector

Reagent	Amount
dsOligo	40 fmol
pYTK050	20 fmol
NEB T4 buffer 10×	1.0 μl
NEB T7 ligase	0.5 μl
NEB BsmBI	0.5 μl
ddH_2_O	to 10 μl

**Table 2:** Golden Gate reaction for cloning gRNA cassette plasmid

Reagent	Amount
gRNA on pYTK050	20 fmol
ConL1 (pYTK003)	20 fmol
ConRE (pYTK072)	20 fmol
AmpR-ColE1 (pYTK083)	20 fmol
NEB T4 buffer 10×	1.0 μl
NEB T7 ligase	0.5 μl
NEB BsaI	0.5 μl
ddH2O	to 10 μl

**Table 3:** Golden Gate reaction for cloning CRISPR plasmid

Reagent	Amount
gRNA cassette plasmid with connectors	20 fmol
Cen6-Ura cassette*	20 fmol
Cas9 plasmid (pYTK036)	20 fmol
NEB T4 buffer 10×	1.0 μl
NEB T7 ligase	0.5 μl
NEB BsmBI	0.5 μl
ddH_2_O	To 10 μl

* Cen6-Ura is constructed by assembling YTK plasmids (008, 047, 073, 074, 081, and 084).
